# Comparative Metabolic Responses Induced by Pyridine and Imidazole in *Blakeslea trispora*

**DOI:** 10.3389/fbioe.2019.00347

**Published:** 2019-11-25

**Authors:** Yang Liu, Xiang-yu Li, Shu-huan Lu, Chao Yu, Yu-zhou Zhang, Zhi-ming Wang, Jian-ming Yao

**Affiliations:** ^1^Biotechnology Center, Institute of Plasma Physics, and Hefei Institutes of Physical Science, Chinese Academy of Sciences, Hefei, China; ^2^University of Science and Technology of China, Hefei, China; ^3^CABIO Biotech (Wuhan) Co. Ltd., Wuhan, China

**Keywords:** metabolism, lycopene, pyridine, imidazole, *Blakeslea trispora*

## Abstract

Lycopene cyclase needs to be inhibited by the blockers like pyridine or imidazole in the lycopene accumulation of *Blakeslea trispora*. This work investigated how pyridine and imidazole impacted the basal metabolism of *B. trispora*, the results helped us understand how they could affect the lycopene production and application, and see the metabolic risks from different inhibitors. In this study, the highest yield of lycopene with pyridine was obtained at 176 mg/L without amino acids supplement, and got more lycopene at 237 mg/L adding tyrosine, lysine, proline all together as 0.01 mol/L each in fermented broth. GC-MS and Principal Component Analysis (PCA) were used to find that amino acids, fatty acids, organic acids including phosphoric acid, carbon source and imidazole derivatives played the most important roles in lycopene fermentation with imidazole, differently, fatty acids, carbon source, and pyridine derivatives were more significant in the pyridine process and it was remarkable that the residual of both blockers' derivatives would bring the potential risks on applications of lycopene products. Predominantly, durene met 0.35 mg/g DCW with imidazole and piperidine formaldehyde attained 0.24 mg/g DCW with pyridine after the end of lycopene fermentations.

## Introduction

*Blakeslea trispora* is industrially viewed as a natural resource of carotenoids, including β-carotene (BC) and lycopene (LYC). Lycopene is synthesized via mevalonate (MVA) pathway in *B. trispora* (Venkateshwaran et al., 2015). One of the key enzymes in the pathway, lycopene cyclase, is a bifunctional enzyme to catalyze lycopene to β-carotene, and also has phytoene synthase activity (He et al., 2017). Thus, lycopene cyclase needs to be inhibited purposefully by blockers to accumulate lycopene.

Imidazole has been proved as a feasible blocker in industry (López-Nieto et al., 2004), while pyridine had been used as blocker to make the highest yield of lycopene at 39.01 mg/L from Xu et al. (2006) in the published reports. It means metabolic differences analysis between pyridine and imidazole could be made to support the capacity of promoting lycopene by pyridine. More importantly, it is remarkable that imidazole used during fermentation in industry may be found in lycopene at levels below 1 mg/kg (Zofia, 2006), but there is not any information reported about the derivatives of imidazole or any other inhibitor. Thus, this work will help to realize the risk of by-products from pyridine and imidazole and find the characteristics of metabolism in lycopene fermentation after mixing inhibitors. The intracellular micro-molecule is affected by the combination of the media, and the changes of metabolites could response the relationship between biological systems and environmental variations (Jia et al., 2016).

In this work, we exploited GC-MS to detect the variation of micromolecular in the processes of lycopene fermentation with different blockers, which is the one of most recognized methods on metabolism analysis (Dettmer et al., 2007; Papadimitropoulos et al., 2018), and after that, PCA were performed to analyze the data.

In the process of lycopene biosynthesis by *B. trispora*, it was reported that the quantitative analysis of glycerol, alcohol and phytoene was made, etc. (Fiehn et al., 2000; Roessner et al., 2001). In this research, some conventional metabolites and derivates of blockers were investigated and it was found a serious discrepancy between the processed of pyridine and imidazole.

## Methods

### *B. trispora* and Fermentation Conditions

*Blakeslea trispora* CBOM2014378(+) and CBOM2014379(−) were used as the fermentation strains.

The fermentation was performed at 28°C, pH 7.2(by glucose feeding) and 30% dissolved oxygen (DO), and got the maximum production of carotenoids at 96 h. To get lycopene, we added 2 g/L of pyridine in the fermentation at 30 h or supplemented 1 g/L of imidazole at 30 h. According to the concentration of amino acids detected in BC fermentation, 0.01 mol/L of tyrosine, lysine, and proline were added back separately and together (Supplementary Data Sheet 1).

### GC-MS Conditions and Principal Component Analysis (PCA)

According to the analysis methods of metabolism from Lisec et al. (2006) and Simon-Manso et al. (2013), GC-MS method was used in this research. At first, *B. trispora* mycelia were quickly gathered at culturing points of 24, 36, 48, 60, 72, 84, and 96 h. Then the metabolites of these samples were extracted by chemical methods after obstructing metabolism. The metabolites were derivatized using methoxyamine hydrochloride and MSTFA. At last, the derivatized metabolites were detected by GC-MS, and data were identified as chemicals and treated through PCA (Supplementary Data Sheet 2).

## Results

### Imidazole Promoted More Lycopene Accumulation Than Pyridine Did in *B. trispora*

**Figure 1:**
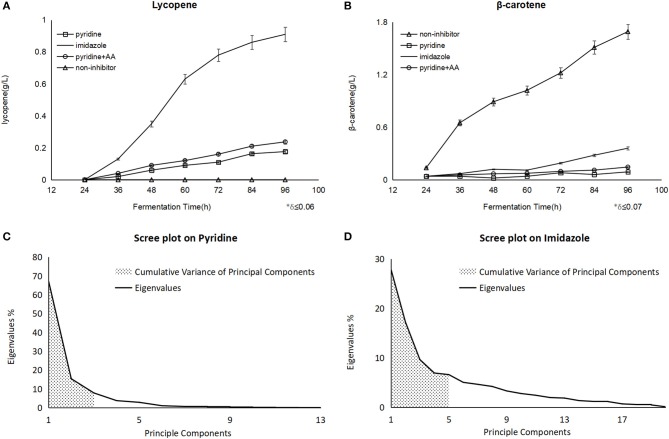
The different yield of carotenoids on the different fermentation and scree plot figures on different blockers were analyzed by PCA. **(A)** Showed lycopene with pyridine had an improvement after the amino acids (AA) addition. **(B)** Showed the same trend on β-carotene. Principle components meant the number of components classified by PCA, and eigenvalues meant the influence from every component on each principle component, the bigger eigenvalue was, the larger impact was. The first three principle components on pyridine were embraced and the sum of their eigenvalues (shadow area) showed us the representativeness of the components **(C)**, as it was explained 90.5% of the whole components data, the same explanation as above, while there were five ones on imidazole **(D)**, which explained 68.2% (shadow area) of the whole components data.

After the pyridine addition, the lycopene production of 176 mg/L was obtained at 96 h in fermentation and 93.7 mg/L β-carotene was detected, while a dramatic improvement of lycopene to 237 mg/L at 96 h as well as 111.4 mg/L β-carotene when some amino acids like tyrosine, lysine, proline were supplemented at 40 h. As the positive control, 911 mg/L of lycopene was accumulated at 96 h with imidazole (Figure 1A), and there was 1.72 g/Lβ-carotene instead of lycopene found in the non-inhibitor process as the negative control (Figure 1B). Nevertheless, the biomass shows no significant change whether blockers were added or not.

### Analysis of Metabolites by PCA

During the lycopene fermentation of *B. trispora*, the whole of 38 and 13 leading metabolites were identified in virtue of imidazole and pyridine addition in that order. The major classes of these ingredients took account of carbohydrate, amino acids, fatty acids, organic acids, and blockers' derivatives. Even though comparable kinds of metabolites were found in the both of blockers' fermentations, their appearances varied expressively. Most of the metabolites were confirmed by KEGG Databases.

In the lycopene process with pyridine, PCA revealed that the Total Variance explained by the first three factors were 67.3, 15.4, and 7.8%, respectively, and they could explain 90.5% of the total variance (Figure 1C). The three factors were labeled:

1. Palmitic acid and pyridine derivatives
2. Carbon source
3. Octadecanoic acid

In the lycopene process with imidazole, PCA revealed that the Total Variance explained by the first five factors were 27.9, 17.2, 9.6, 6.9, and 6.6%, respectively, and they could explain 68.2% of the total variance (Figure 1D). The five factors were labeled:

1. Amino acids
2. Fatty acids
3. Organic acids metabolism and phosphoric acid
4. Carbon source
5. Benzene and its derivatives

The two processes are obviously distinguished by amino acid metabolism, lipid metabolism and blockers' derivates as shown above. In the β-carotene fermentation without inhibitors, amino acids metabolism was vigorous, a dozen of amino acids were detected, but it was partly blocked with imidazole, and totally stagnated with pyridine. Both of lipid metabolism and blocker derivation were found to be remarkable in the lycopene fermentation.

The result indicated that both of pyridine and imidazole could inhibit the lycopene cyclase, and pyridine fermentation mode had made much less metabolic intermediates detected, which meant most metabolisms were inhibited, amino acid metabolism was totally blocked, and only linoleic acid and the pyridine derivatives were kept in the rather high level relatively. Imidazole made a higher metabolic level of organic acids, and some of amino acids were inhibited seriously.

## Discussion

### Variations of Amino Acids

**Figure 2:**
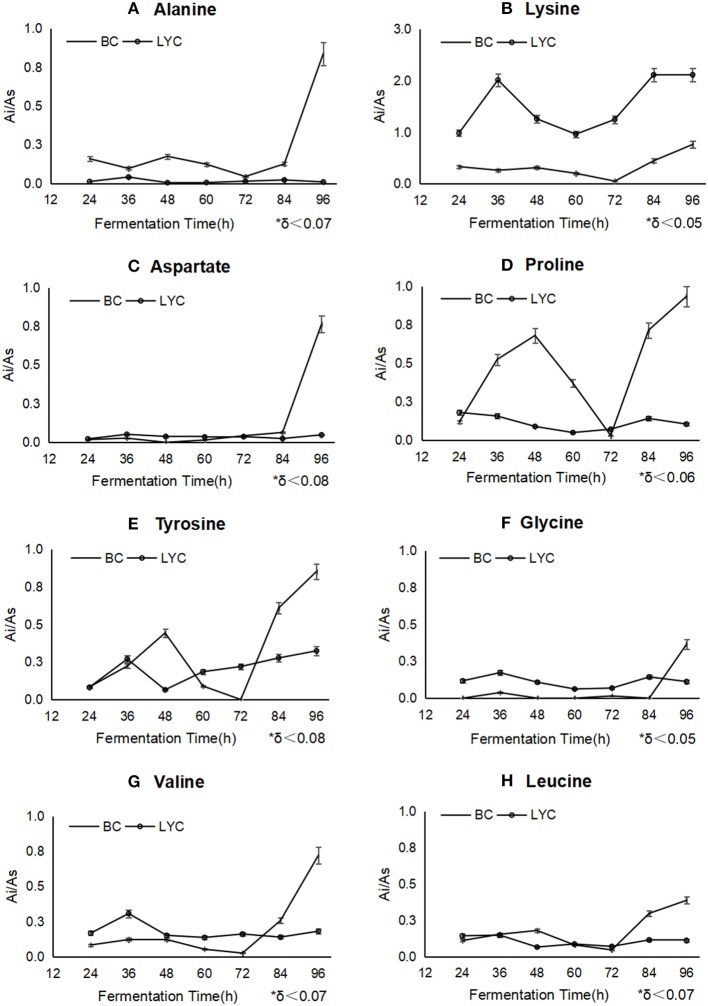
The change of different amino acids during the fermentations betweenβ-carotene mode and lycopene mode. BC stood for β-carotene mode without any inhibitor, and LYC stood for lycopene mode with imidazole. Ai/As showed relative concentration of every amino acid basing on internal label. Alanine **(A)**, proline **(D)**, and tyrosine **(E)** were inhibited after imidazole addition, lysine **(B)**, glycine **(F)**, and valine **(G)** were stimulated to a higher concentration, aspartate **(C)** and leucine **(H)** had almost no change during the process.

As showed in Figure 2, the intracellular content levels of aspartate (Figure 2C), tyrosine (Figure 2E), valine (Figure 2G), and leucine (Figure 2H) were close to those of the control group (β-carotene fermentation by *B. trispora* without blockers) and stabilized during fermentation, the amounts of lysine (Figure 2B), proline (Figure 2D), and tyrosine (Figure 2E) were innocently reduced from 36 to 72 h, and significantly increased from 72 to 96 h, changing from 0.01 to 2 mg/g. The most probable explanation was that alanine reacted with glyoxylate by aminotransferase to produce pyruvate and glycine (Okuno et al., 1982). Proline could be used as precursor to biosynthesize glyoxylate and pyruvate, while pyruvate metabolism, glycolysis, and citrate cycle would be flowed to the lysine biosynthesis by homoaconitase (Scholtz and Schuster, 1986; Niot et al., 2009). The metabolic pathways of aspartate, tyrosine, proline, valine, and leucine can participate in the protein biosynthesis or fatty acids through TCA circulation, therefore, they ensured the synthesis of structure protein to a certain extent in fungi, so the mycelia were close to its natural form. Nevertheless, amino acids metabolism was totally inhibited with pyridine addition, and the mycelia were fragile and loose.

It was verified that the lack of amino acid metabolism would inhibit the biosynthesis of lycopene. lysine, proline, and tyrosine were picked up to add back in the culture medium after pyridine addition, since they were detected at a higher concentration in cells (Figures 2B,D,E). The result of lycopene improvement was found to demonstrate that lysine, proline, and tyrosine surely conduce to the biosynthesis of lycopene (Figure 1A) up to 237 mg/L from 176 mg/L, so did β-carotene (Figure 1B) to a less extent increasing 18.9%. It must be emphasized that the mixed addition of these amino acids had a better effect than the single addition of any one of them, which probably meant the more amino acids would partly recover the biosynthesis or activity of some key enzymes on carotenoid biosynthesis. Pyridine seriously inhibited the amino acids metabolism of *B. trispora*, but most of amino acids were still detected under the imidazole condition. It indicated that pyridine inhibited the protein biosynthesis including many enzymes on carotenoids biosynthesis, and it was the reason why lycopene yield was too low to use pyridine in industry, but the supplement of cheap amino acids would be a feasible solution.

### Variations of Phosphoric Acid and Fatty Acids

**Figure 3:**
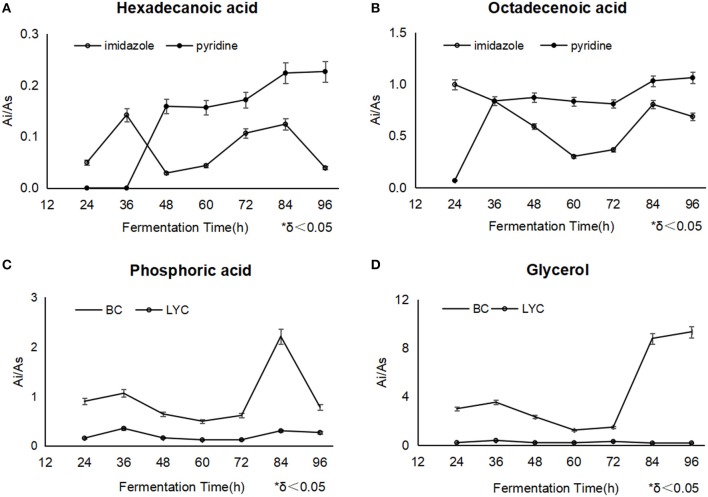
The changes of hexadecanoic acid **(A)** and octadecanoic acid **(B)** between different fermentations with imidazole and pyridine, the changes of phosphoric acid and glycerol between β-carotene fermentation **(C)** and lycopene fermentation with imidazole **(D)**.

Phosphoric acid usually played an important role in the adjustment of signal transduction route, and participated in converting from ADP to ATP as along as oxidizing citrate in TCA cycle and glycolysis reactions (Ding et al., 2009). the detected phosphoric acid fell obviously after the imidazole addition at 36 h, corresponding to the normal process without blockers (Figure 3C), phosphoric acid was accumulated as the high-level TCA cycle involved (Zhou et al., 2009), while in the processes with pyridine, it was thought that phosphoric acid possibly neutralized with it, besides normal metabolic response.

Hexadecanoic acid displayed a similar variation trend during the both fermentation of different blockers. But in the pyridine process, linoleic acid and octadecanoic acid were relatively kept in a higher level from the addition point to the end, compared with imidazole process, which meant mycelia accumulated more oil (Figure 3A). Thus, the mycelia under the pyridine condition looked obese.

It was widely reported that fatty acids promoted β-carotene and lycopene synthesis (Singh et al., 2018; Ma et al., 2019). Octadecanoic acid could be utilized in the conversion to lycopene, differently, imidazole obviously had a less inhibition on the enzymes of lycopene biosynthesis except lycopene cyclase, while pyridine had a total toxicity on the biosynthesis system since such a discrepancy of lycopene results mentioned as above (Figure 3B). In all, the level of fatty acids throughout the whole process showed us that imidazole promoted fatty acids' availability and pyridine just stored up the oil without a high-efficient utility.

It was indicated that TCA cycle was kept in a much lower level because of the blockers' addition as fumarate and malate were barely found, but octadecanoic acid was maintained a relatively higher level under the both of inhibitors, thus oil including fatty acids should be a better choice of carbon source in the lycopene process. The fatty acids, especially octadecanoic acid obviously had been promoted to a higher metabolic level with blockers, meanwhile, hexadecanoic acid were used marginally.

### Variations of Carbon Source

It was found that the intracellular glucose was in a very low concentration in the both fermentations. Imidazole made more glycerol, and reached 9.41 μg/mg DCW at 96 h (Figure 3D). It was regarded that the intracellular glycerol played an primary role as a compatible solute to adjust the permeability of cell membranes by triggering the high osmolarity glycerol (HOG) pathway (Hohmann, 2002), which motivated cells to comply with the hyperosmotic stress conditions (Dihazi et al., 2004; Petelenz-Kurdziel et al., 2013; Sabir et al., 2017).

The level of glycerol in the lycopene fermentation was relatively high during the whole process. The most likely explanation is that intracellular glycerol touched a great intensity speedily by the HOG pathway to guard cells from withering at the high sugar concentration, and the hyperosmotic stress declined along with the depletion of glucose during the fermentation progress. Glycerol is also an intermediate from oil metabolism, which was consumed in the middle stage of fermentation. Afterwards, metabolic collapse might be happened in the apoptosis phase, and the unconsumed glycerol accumulated gradually. It was likely that pyridine weaken the HOG pathway to some extent.

What's more, it was also found that galactose was produced in the imidazole process, and only inositol was produced in the pyridine process slightly.

### Variations of Blockers' Derivatives

**Figure 4:**
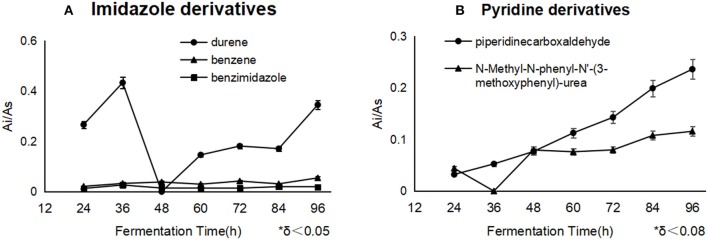
The different derivatives of blockers accumulated as the fermentation processes. Durene and piperidinecarboxaldehyde were the essential byproducts of imidazole and pyridine separately. Durene **(A)** showed a much higher concentration than other derivatives in the cells with imidazole, and pyridine derivatives **(B)** constantly accumulated during the whole process.

Most importantly, the derivatives of the blockers would pose a threat to the applied security, which was not found in the reports before. In this work, benzene, benzimidazole, and durene were derived from imidazole, and piperidinecarboxaldehyde and N-Methyl-N-phenyl-N'-(3-methoxyphenyl)-urea were derived from pyridine. Besides, durene and piperidine formaldehyde were accumulated constantly in cells, in the end of fermentation, durene met 0.35 μg/mg DCW (Figure 4A), and piperidine formaldehyde was attained to 0.24 μg/mg DCW (Figure 4B). The derivatization reaction of benzimidazole referred to 5-hydroxybenzimidazole synthase, it catalyzed a complex oxygen-dependent conversion of reduced flavin mononucleotide to form 5,6-dimethylbenzimidazole. The C-2 of 5,6-dimethylbenzimidazole is derived from C-1′ of the ribityl group of FMNH2 and 2-H from the ribityl 1′-pro-S hydrogen. While D-erythrose 4-phosphate has been shown to be one of the byproducts, the nature of the other product(s) has not been verified yet (Wang and Quan, 2011; Collins et al., 2013) and benzimidazole would be the precursor of Ensulizole. It was noticed that benzene could be reacted to phenol and catechol by phenol 2-monooxygenase, nevertheless, durene, piperidinecarboxaldehyde and N-Methyl-N-phenyl-N′-(3-methoxyphenyl)-urea should be products in some unknown reactions, which were not found or reported.

In the security risk, durene brought the slight oral toxicity (Lynch et al., 1978), hematotoxicity from exposure to benzene occurred at air levels of 1 ppm or less and may be particularly evident among genetically susceptible subpopulations (Lan et al., 2004), Benzimidazoles display a broad spectrum of potential pharmacological activities and are present in a number of pharmacologically active molecules such as Albendazole and Mebendazole (Srikanth et al., 2011), while the other derivatives even had no toxicity information.

It was noticed that imidazole gave rise to some risky derivatives such as durene, benzene and benzimidazole, and pyridine helped to accumulate the derivatives including Piperidinecarboxaldehyde and N-Methyl-N-phenyl-N'-(3-methoxyphenyl)-urea. All of these derivatives were first found as metabolites during the lycopene fermentation, and they were not supervised or controlled yet during the present lycopene industry process, additionally, durene and benzene could not be eliminated by water solvent, which means the residue of them should enrich in lycopene oil and threaten the application security. Above all, the suitable strategy is to use less imidazole as the inhibitor throughout the further fermentation optimization, or to select the high-sensitive strain on imidazole. In the process of fermentation, more oils could be used as carbon source after imidazole addition. Fundamentally, it is the better direction to find natural inhibitor like some alkaloids which could be metabolized.

Moreover, additional work should be needed to figure out how to proficiently control metabolism flow of *B. trispora* under the multifaceted production fermentation situations with studies of lipidomic and proteome, in order to improve the lycopene productivity of the manufacturing strain of *B. trispora*.

## Data Availability Statement

The raw data supporting the conclusions of this manuscript will be made available by the authors, without undue reservation, to any qualified researcher.

## Author Contributions

YL analyzed the data in results sections and drafted the manuscript with XL together. XL and CY did the fermentation experiments. SL and YZ treat the samples and revised the GC-MS analysis parts of compounds in methods. ZW and JY designed the project and modified the manuscript.

### Conflict of Interest

YL, XL, SL, CY, and ZW were employed by the company CABIO Biotech (Wuhan) Co. Ltd. The remaining authors declare that the research was conducted in the absence of any commercial or financial relationships that could be construed as a potential conflict of interest.
