# Supplementary material for: Comparative Metabolic Responses Induced by Pyridine and Imidazole in Blakeslea trispora
Source: Front Bioeng Biotechnol. 2019 Nov 25;7:347. doi: 10.3389/fbioe.2019.00347 (PMC6886401; doi:10.3389/fbioe.2019.00347)
Supplement: Supplementary file 1 [file Data_Sheet_1.docx]

Fermentation Conditions

***B.trispora* and fermentation conditions**

*B.trispora* CBOM2014378(+) and CBOM2014379(-) were used as the fermentation strains.

Spores of strain (+) and strain (-) were distinctly cultivated on potato extract slope medium, which included glucose 2%, potato extract 20%, agar 2.5%, sporulation incubating at 27℃ for 6 days, afterwards rinsing the slope medium by the purified water to gather the spores. The spores were dispensed into the seed incubation medium and the seed was dispensed into the fermentation medium in a 50L cylinder, the component parts of mediums were showed in Table 1.

**Table. 1 The composition of mediums**

| Medium | Composition (g/L) | | | | | | |
| --- | --- | --- | --- | --- | --- | --- | --- |
|  | Glucose | Yeast powder | Soybean meal | Linseed oil | KH2PO4 | MgSO4 | Starch |
| Seed | 20 | 15 | 35 | 10 | 0.7 | 0.1 | \ |
| Fermentation | 10 | 20 | 20 | 40 | 1 | 0.3 | 30 |

Spore suspensions (about 5×10^4^ spores per 1mL) of mating type (+) and (-) were singly injected into 150mL seed culture(pH 7.5) to get the primary seed cells at 27℃, 200r/min for 44h, and then each transferred into 20L pre-culture medium as the secondary seed, which were cultivated without pH control at 27℃,1vvm,and 250 r/min for 40h. Finally, 1L (+) seed and 6L (-) seed were vaccinated together into 25L of fermentation medium (pH 7.5). The fermentation was performed at 28℃, pH 7.2(by glucose feeding) and 30% dissolved oxygen (DO), and got the maximum production of carotenoids at 96h. To get lycopene, we added 2g/L of pyridine in the fermentation at 30h or supplemented 1g/L of imidazole at 30h.

To verify the effect of the amino acids, according to the results of GC-MS on negative control group, lysine, proline and tyrosine were supplemented as 0.01mol/L separately and all together at 40h in the fermentation, after pyridine’s addition.
