# Supplementary material for: Comparative Metabolic Responses Induced by Pyridine and Imidazole in Blakeslea trispora
Source: Front Bioeng Biotechnol. 2019 Nov 25;7:347. doi: 10.3389/fbioe.2019.00347 (PMC6886401; doi:10.3389/fbioe.2019.00347)
Supplement: Supplementary file 2 [file Data_Sheet_2.docx]

GC-MS Detection

**Samples and extraction of metabolites**

At first, *B.trispora* mycelia were quickly gathered at culturing points of 24h, 36h, 48h, 60h, 72h, 84h and 96h.Then the washed samples were immersed into the cold (-20℃) 60% methanol (v/v) to obstruct metabolism instantly. Afterwards, the mycelia were collected and dropped into liquid nitrogen after being washed and compressed dry to quench the metabolism of cells, mycelia were pulverized to fines by grinder. Secondly, put sample powder (100 mg) into the extraction buffer made up of methanol/chloroform/water (1.5:2.5:1, v/v/v, -20℃, 5 mL) (Koning et al., 1992; Ming-Zhu Ding et al., 2009). The Deuterium labeled Succinic acid (succinic-2,2,3,3-d4 acid) was put in the samples in moderation as the interior label to revise the tiny alteration happened in sample and data manipulation (Jian Zhou et al.2011). After a centrifugation at 6,000r/min at -20℃ for 8 min, the cell pellets were refined with methanol aqueous solution (1mL,1:1, v/v, -20℃). The mixture contained the all hydrophilic metabolites which needed to be frozen under a low temperature (-80℃). At last, every sample had five replicates.

**Metabolites derivatization**

The obtained metabolites were dually carried out by derivatization for GC-MS testing (Drupad et al., 2016). Primarily, dissolving the metabolites with methoxyamine hydrochloride in pyridine for oximation (20mg/mL, 5µl) and hatching at 40℃ for 80 min. Formerly, further derivatization for 90 min by adding 80µl MSTFA was made for trimethylsilylating the polar groups at 40℃ (Shuhuan Lu et al., 2012). Lastly, equilibrizing the samples to room temperature before injection, after centrifugation at 12000r/min for 3 min.

**GC-MS test**

The GC-MS architecture contained a 7890A GC system equipped with a 5975c MS (Agilent, USA), a DB-5MS column (30 mm×0.25 mm I.D., df 0.25 µm), a flame ionization detector (FID), a Deans switch transfer device, and an autosampler. The carrier gas appointed helium as usual. The sample (1 µl) poured through injector according to a split ratio of 50:1 when the injector temperature reached 290℃. The work-flow was kept going under the invariable pressure of 58 kPa in column, and the column’s temperature was conducted at 70 ℃ maintaining 3 min, subsequently rose up 5 ℃ every minute to 290 ℃ and staying 15 min. A 70eV electron impacted to make ions. Mass scan ranged from 50 m/z to 800 m/z with the rate of 20 scans/s.

**Data treatment**

Agilent MSD chem-station was applied for the analysis of mass spectral peaks. The S/N values were chosen by the software for quantification, which means the signal/noise value lower than 30 could be rejected. The retention time and m/z determined the identiﬁed metabolites by comparing in NIST database. Each peak area corresponding to the detected materials was regularized by internal label, as well as the relative abundance of metabolites was qualified to DCW of the sample. Peaks with similarity superior than 80% were confirmed as materials. For relative quantification and multivariate statistical analysis, all of data were standardized to the internal label on the same chromatogram (Wang et al., 2014). For all the samples obtained from the fermentation processes of nicotine discrepancy, every normalized result finally calculated the average of five individual replicates, which were arranged to carry out PCA.

**References:**

Koning, W. de, & Dam, K. van. (1992). A method for the determination of changes of glycolytic metabolites in yeast on a subsecond time scale using extraction at neutral pH. *Analytical Biochemistry*, 204(1), 118–123.

Ding, M. Z., Cheng, J. S., Xiao, W. H., Qiao, B., & Yuan, Y. J. (2009). Comparative metabolomic analysis on industrial continuous and batch ethanol fermentation processes by GC-TOF-MS. *Metabolomics*, 5(2), 229.

Zhou, J., Ma, Q., Yi, H., Wang, L., Song, H., & Yuan, Y. J. (2011). Metabolome profiling reveals metabolic cooperation between Bacillus megaterium and Ketogulonicigenium vulgare during induced swarm motility. Appl. Environ. Microbiol., 77(19), 7023-7030.

Lu, S., Wang, J., Niu, Y., Yang, J., Zhou, J., & Yuan, Y. (2012). Metabolic profiling reveals growth related FAME productivity and quality of Chlorella sorokiniana with different inoculum sizes. Biotechnology and Bioengineering, 109(7), 1651-1662.

Wang, X., Jin, M., Balan, V., Jones, A. D., Li, X., Li, B. Z., Dale, B. E. & Yuan, Y. J. (2014). Comparative metabolic profiling revealed limitations in xylose‐fermenting yeast during co‐fermentation of glucose and xylose in the presence of inhibitors. Biotechnology and bioengineering, 111(1), 152-164.
